# First-Trimester Neutrophil Percentage-to-Albumin Ratio Adds Meaningful Additive Prognostic Utility for Early-Onset Preeclampsia: A Nested Case-Control Study

**DOI:** 10.3390/jcm15155986

**Published:** 2026-07-31

**Authors:** Mustafa Koçar, Özlem Ulaş

**Affiliations:** 1 Department of Obstetrics and Gynecology, Kütahya City Hospital, 43100 Kütahya, Turkey; 2 Department of Obstetrics and Gynecology, Kütahya Health Sciences University, 43100 Kütahya, Turkey; ozlemulas.dr@gmail.com

**Keywords:** early-onset preeclampsia, neutrophil percentage-to-albumin ratio, first trimester, risk prediction, inflammation, maternal biomarkers

## Abstract

**Background:** Early-onset preeclampsia (EO-PE) remains a major cause of maternal and perinatal morbidity and mortality, and early risk stratification is still limited by the lack of simple and widely accessible biomarkers. The neutrophil percentage-to-albumin ratio (NPAR) has emerged as a composite marker reflecting systemic inflammation and endothelial dysfunction. **Methods:** In this retrospective, nested case–control study (*n* = 1176), we evaluated the predictive performance of first-trimester NPAR for EO-PE (*n* = 168) versus frequency-matched normotensive controls (*n* = 840) and its incremental value beyond established clinical and hemodynamic parameters. Multivariable models were adjusted for gestational age at sampling, and internal validation was performed via bootstrapping. **Results:** NPAR demonstrated superior discrimination (AUC = 0.848) compared with NLR (AUC = 0.629) and MAP (AUC = 0.614) (*p* < 0.001 for both). At the optimal cutoff of 16.52, NPAR showed a sensitivity of 81.5% (95% CI: 74.8–87.1%) and a specificity of 76.0% (95% CI: 72.9–78.9%), yielding a prevalence-adjusted positive predictive value (PPV) of 6.5% and a negative predictive value (NPV) of 99.5% based on Bayes’ Theorem. In multivariable analysis, NPAR remained independently associated with EO-PE (aOR 2.25, 95% CI 1.81–2.80; *p* < 0.001, per 1 SD increase). The addition of NPAR significantly improved model performance, increasing the AUC from 0.612 to 0.830 (DeLong *p* < 0.001), with robust bootstrap stability. **Conclusions:** First-trimester NPAR stands out as a potential independent clinical risk vector for early-onset preeclampsia and may offer meaningful additive prognostic utility beyond conventional clinical parameters. Given its low cost and routine availability, it may represent an accessible framework for initial frontline triage in early pregnancy risk stratification, particularly in resource-limited clinical settings where advanced multi-marker screening algorithms are unavailable. Rigorous external validation in prospective, multicenter longitudinal cohorts remains an absolute prerequisite before routine implementation in clinical management pathways.

## 1. Background

Preeclampsia (PE) is a pregnancy-specific hypertensive disorder and remains a leading cause of maternal and perinatal morbidity and mortality worldwide [1]. Affecting approximately 2–5% of pregnancies, PE is associated with a wide spectrum of adverse maternal and neonatal outcomes [2]. Despite well-established diagnostic criteria, early prediction of PE remains a major unmet clinical need.

Accumulating evidence indicates that systemic inflammation plays a central role in the pathogenesis of PE. Exaggerated inflammatory responses contribute to endothelial dysfunction, oxidative stress, and placental hypoperfusion, which are key mechanisms in disease development [3,4,5]. Inflammatory cells accumulate within the vascular compartment, disrupting endothelial homeostasis and promoting placental insufficiency [6,7,8,9]. Accordingly, both clinical and experimental studies have consistently demonstrated a close association between inflammatory pathways and the occurrence of PE [10,11,12].

Preeclampsia is now widely regarded as a multifactorial pregnancy complication in which placental-derived factors trigger widespread maternal inflammation and endothelial dysfunction, involving both innate and adaptive immune responses [13]. Among the inflammatory pathways implicated in PE, neutrophil-mediated mechanisms appear to be particularly relevant. Increased neutrophil number and activation, delayed apoptosis, and sustained inflammatory signaling contribute to prolonged endothelial injury and vascular dysfunction in PE [14].

In clinical practice, several hematological indices reflecting systemic inflammation have been investigated as potential biomarkers for PE. The neutrophil-to-lymphocyte ratio (NLR) has been associated with disease severity, particularly in severe forms of PE [15]. Notably, a large meta-analysis including 3982 pregnancies confirmed significantly higher NLR values in women with PE, especially in severe disease, supporting a role for neutrophil-driven inflammation in disease progression [15]. However, most available evidence is derived from case–control studies, and data regarding early prediction, optimal timing, clinically relevant cut-off values, and incremental predictive performance remain limited. Currently, well-established first-trimester prediction algorithms integrated by the Fetal Medicine Foundation (FMF)—which combine maternal characteristics with mean arterial pressure (MAP), uterine artery pulsatility index (UtA-PI), and placental growth factor (PlGF)—demonstrate optimal performance in screening for early-onset preeclampsia, as validated globally by Poon et al. [16]. However, the absolute necessity for high-cost biochemical assays and expert-dependent Doppler ultrasonography restricts their widespread implementation in low-resource or community-based primary healthcare settings. This underlines the clinical necessity of exploring rapid, universally standardized, and cost-effective alternatives like NPAR to serve as frontline risk-stratification parameters.

Neutrophil-related parameters and serum albumin levels have long been used as indicators of inflammatory status and nutritional condition [17,18]. These findings provide a biological rationale for combining neutrophil-related parameters with albumin-based measures into a composite index that reflects the integrated inflammatory–endothelial axis of the disease.

More recently, the neutrophil percentage-to-albumin ratio (NPAR) has emerged as a novel composite inflammatory marker. NPAR integrates neutrophil-driven inflammation and albumin-related endothelial dysfunction into a single, clinically accessible biomarker. Outside the obstetric field, NPAR has demonstrated strong prognostic value in various clinical settings, including cardiogenic shock, acute kidney injury, myocardial infarction, and coronary artery disease [19,20,21,22]. In obstetric pathology, Biçer et al. evaluated NPAR in pregnancies complicated by preeclampsia and reported its association with the disease, highlighting NPAR as a potentially valuable inflammatory marker [23]. Nevertheless, its predictive performance in early pregnancy and, importantly, its incremental predictive value beyond established clinical and hemodynamic parameters have not been systematically evaluated.

Despite advances in understanding the inflammatory and vascular mechanisms underlying PE, important gaps persist in early disease prediction. There remains a need for rapid, inexpensive, and widely accessible biomarkers that can be integrated into routine clinical practice.

Therefore, the aim of the present study was to evaluate the neutrophil percentage-to-albumin ratio as a novel composite inflammatory biomarker and to determine its predictive performance for early-onset preeclampsia utilizing a rigorous nested case–control design. In addition, we specifically investigated whether NPAR provides incremental predictive value beyond established clinical and hemodynamic parameters within a multivariable prediction model.

To our knowledge, this study is among the first to evaluate the incremental predictive value of first-trimester NPAR within a clinically applicable multivariable model for early-onset preeclampsia, supported by a well-balanced, frequency-matched control population.

## 2. Materials and Methods

### 2.1. Study Design and Population

This retrospective, nested case–control study was conducted at a tertiary referral center and included data collected over a seven-year period. Medical records of singleton pregnancies that underwent routine first-trimester (11 + 0 to 13 + 6 weeks of gestation) laboratory evaluation and subsequently delivered at our clinic during the study period were retrospectively reviewed.

Pregnancies complicated by early-onset preeclampsia (EO-PE, *n* = 168) and late-onset preeclampsia (LO-PE, *n* = 168) were identified. To minimize confounding from baseline maternal factors and enhance statistical power, a strict 1:5 frequency matching protocol was utilized for control selection. For each preeclampsia case, five normotensive controls (*n* = 840) were randomly selected from the cohort of women who delivered at the same center during the identical time period. The control group consisted of pregnancies that remained normotensive throughout gestation and had no diagnosis of hypertensive disorders of pregnancy. This approach was used to minimize temporal, selection, and referral bias.

The study was conducted in accordance with the principles of the Declaration of Helsinki and was approved by the local institutional ethics committee (approval number: 2025/11-01, date: 15 September 2025).

Early-onset preeclampsia (EO-PE) was defined as preeclampsia diagnosed and requiring delivery before 34 weeks of gestation.

### 2.2. Inclusion and Exclusion Criteria

Singleton pregnancies with available first-trimester complete blood count parameters and serum albumin measurements obtained strictly during the routine first-trimester aneuploidy screening window (between 11 + 0 and 13 + 6 weeks of gestation) were eligible for inclusion. Gestational age was determined based on first-trimester ultrasonographic measurements (crown-rump length [CRL] measuring between 45 mm and 84 mm, corresponding to 11 + 0 and 13 + 6 weeks of gestation) to ensure absolute chronological and physiological standardization. Pregnancies with chronic hypertension, pregestational diabetes mellitus, renal or hepatic disease, autoimmune disorders, hematologic disease, acute or chronic infection at the time of blood sampling, multiple gestations, or fetal structural or chromosomal anomalies were excluded. Pregnancies with missing laboratory data, incomplete medical/delivery records, or those lost to follow-up before delivery were also excluded. Furthermore, participants receiving medications that could affect inflammatory markers at the time of first-trimester sampling, such as systemic corticosteroids or immunosuppressive therapies, were excluded from the final selection. Regarding missing data management, a listwise deletion approach was utilized; patients with missing baseline covariates or incomplete primary laboratory outputs (representing <2% of the initial eligible population) were excluded prior to the 1:5 frequency-matching process, ensuring a completely intact and fully verified final analytic sample.

### 2.3. Data Collection

Maternal demographic characteristics, obstetric history, parity, smoking status, gestational diabetes, aspirin use, blood pressure measurements, and pregnancy outcomes were obtained from electronic medical records. Mean arterial pressure (MAP) was calculated using the standard formula: MAP = (systolic blood pressure + 2 × diastolic blood pressure)/3. Importantly, documented aspirin use strictly reflects baseline maternal therapy initiated prior to or at the exact time of the first-trimester blood sampling, ensuring it represents a pre-existing independent covariate rather than a post-screening clinical intervention.

### 2.4. Laboratory Measurements

Peripheral venous blood samples were obtained at the exact time of the maternal first-trimester screening visit, corresponding to a tightly regulated gestational age of 11 to 13 + 6 weeks. Complete blood count parameters, including neutrophil percentage, were analyzed using an automated hematology analyzer (Sysmex XN-1000, Sysmex Corporation, Kobe, Japan). Serum albumin levels were measured using standard biochemical methods via the bromocresol green assay on an automated chemistry analyzer (Roche Cobas c501, Roche Diagnostics, Mannheim, Germany). Both systems were subject to daily internal quality control procedures, maintaining an analytical coefficient of variation (CV) of less than 2.5% to ensure measurement stability and eliminate analytical variability.

The neutrophil percentage-to-albumin ratio (NPAR) was calculated by dividing the neutrophil percentage by the serum albumin concentration (g/dL). The neutrophil-to-lymphocyte ratio (NLR) was calculated as the ratio of absolute neutrophil count to absolute lymphocyte count.

### 2.5. Outcome Definitions

Preeclampsia was defined according to the American College of Obstetricians and Gynecologists (ACOG) criteria as new-onset hypertension after 20 weeks of gestation accompanied by proteinuria or evidence of end-organ dysfunction. To maintain phenotypic precision, preeclampsia was further categorized based on delivery timing: early-onset preeclampsia (EO-PE) was strictly defined as preeclampsia diagnosed and requiring delivery before 34 + 0 weeks of gestation, whereas late-onset preeclampsia (LO-PE) was defined as preeclampsia requiring delivery at or after 34 + 0 weeks of gestation.

### 2.6. Statistical Analysis

Statistical analyses were performed using SPSS version 26.0 (IBM Corp., Armonk, NY, USA) and R software (version 4.3.1). Continuous variables were assessed for normality using visual inspection (histograms and Q-Q plots) and analytical tests (Shapiro–Wilk test). We expressed normally distributed variables as mean ± standard deviation and non-normally distributed variables as median (interquartile range). Group comparisons were conducted using the independent samples *t*-test or Mann–Whitney U test, as appropriate. Categorical variables were compared using the chi-square test or Fisher’s exact test, as appropriate. Receiver operating characteristic (ROC) curve analyses were performed to evaluate the predictive performance of NPAR, NLR, and MAP for early-onset preeclampsia, and the area under the curve (AUC) values were calculated. The optimal clinical cutoff value for NPAR was identified via the maximum Youden Index, and corresponding diagnostic performance metrics—including sensitivity, specificity, positive predictive value (PPV), and negative predictive value (NPV)—were computed with 95% confidence intervals (CI). Comparisons between AUCs were performed using the DeLong method. To construct a clinically stable and epidemiologically sound prediction instrument, all continuous and categorical clinical predictors—including maternal age, body mass index (BMI), parity, smoking status, gestational diabetes (GDM), pre-existing baseline aspirin therapy, and mean arterial pressure (MAP)—were selected strictly a priori based on their absolute clinical relevance, pathophysiological plausibility, and availability at the first-trimester screening visit timeline, thereby intentionally avoiding suboptimal automated variable selection algorithms or univariable screening filtering criteria. To rigorously evaluate potential non-linear relationships between these continuous independent predictors (NPAR, MAP, maternal age, and BMI) and the log-odds of early-onset preeclampsia, a formal Restricted Cubic Splines (RCS) analysis modeled with 4 knots placed at standard percentiles was executed utilizing the ‘rms’ package in R software. Multivariable logistic regression analysis was subsequently performed to identify independent risk factors of early-onset preeclampsia, adjusting for maternal age, body mass index, parity, smoking status, gestational diabetes, and baseline aspirin use, while strictly adjusting for continuous gestational age at blood sampling to control for first-trimester physiological variability and hemodilution. Continuous NPAR values were standardized and modeled per 1 Standard Deviation (SD) increase across the cohort population to optimize clinical interpretability and bedside application. Adjusted odds ratios (aORs) with full raw regression coefficients (β), standard errors (SEs), Wald chi-square statistics, and 95% confidence intervals were reported. Multicollinearity among the predictors was formally assessed using the Variance Inflation Factor (VIF), with a VIF < 2.0 indicating no significant collinearity. In strict accordance with the TRIPOD guidelines framework established by Collins et al. [24], internal validation of the final multivariable prediction model was executed using bootstrapping with 1000 resamples to calculate the optimism-corrected AUC. Model calibration was comprehensively evaluated using the Brier score, calibration slope/intercept, and the Hosmer-Lemeshow goodness-of-fit test. To evaluate the clinical utility and net benefit of the predictive model across various risk thresholds, we performed Decision Curve Analysis (DCA), as originally conceptualized by Vickers and Elkin [25], to calculate the net clinical benefit of incorporating NPAR into the screening pathway. All statistical tests were two-sided, and a *p*-value < 0.05 was considered statistically significant.

Generative Artificial Intelligence (GenAI) Disclosure: During the revision phase of this manuscript, a generative artificial intelligence tool was utilized solely to enhance the linguistic clarity, academic tone, and overall readability of the text. No GenAI applications were employed for study design execution, data collection, statistical data analysis, or primary clinical interpretation.

## 3. Results

### 3.1. Baseline Characteristics and Inflammatory Markers

**Figure 1:**
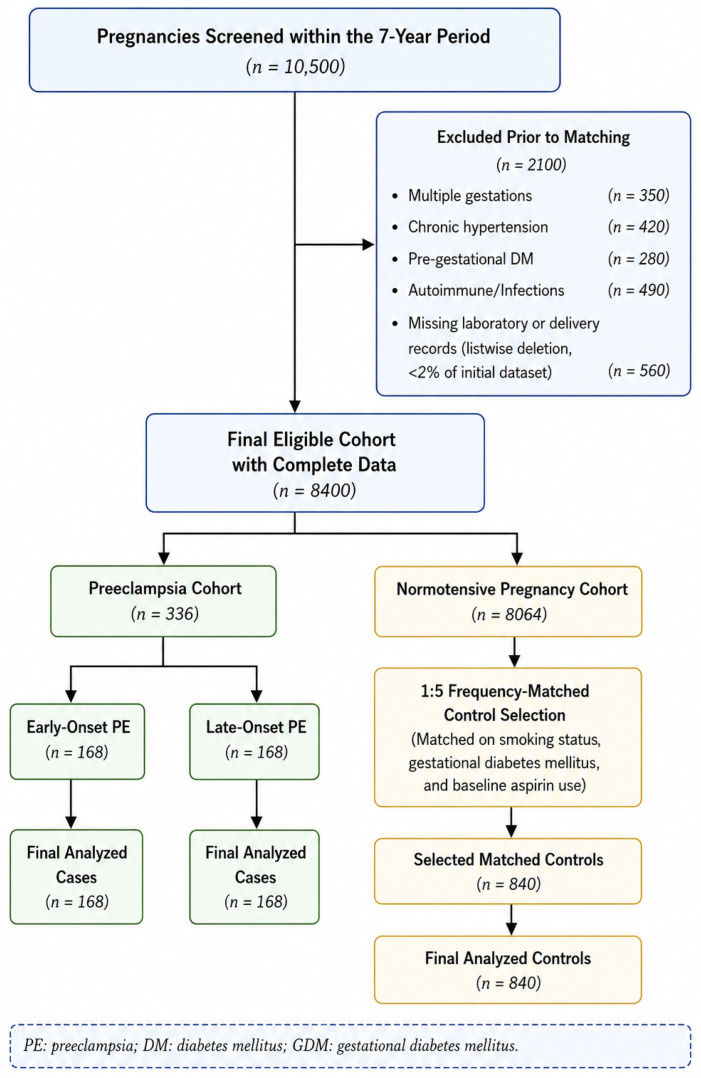
Flow diagram of the participant screening, selection, and frequency-matching process compliant with the STROBE (Strengthening the Reporting of Observational Studies in Epidemiology) statement. The final nested case–control architecture evaluates early-onset preeclampsia (EO-PE) cases against matched normotensive controls in a strict 1:5 ratio. PE: preeclampsia; EO-PE: early-onset preeclampsia; LO-PE: late-onset preeclampsia; DM: diabetes mellitus; GDM: gestational diabetes mellitus.

A total of 1176 pregnant women were included in this nested case–control analysis, comprising 168 early-onset preeclampsia (EO-PE) cases, 168 late-onset preeclampsia (LO-PE) cases, and 840 frequency-matched normotensive controls (Figure 1). Baseline maternal characteristics and first-trimester inflammatory indices comparing EO-PE and controls are presented in Table 1. Maternal age and mean arterial pressure (MAP) were significantly higher in the EO-PE group, whereas body mass index (BMI), parity, smoking status, gestational diabetes, and aspirin use did not differ between groups due to the strict 1:5 frequency matching protocol (all *p* = 1.000 for matched covariates).

The first-trimester peripheral blood samples in both groups were obtained within the scheduled aneuploidy screening window, with an identical timing distribution. The median gestational age at blood sampling was 11.8 weeks (IQR: 11.2–12.5) for the normotensive controls and 11.7 weeks (IQR: 11.1–12.4) for the EO-PE group, demonstrating no statistically significant chronological difference between the cohorts (*p* = 0.425).

**Figure 2:**
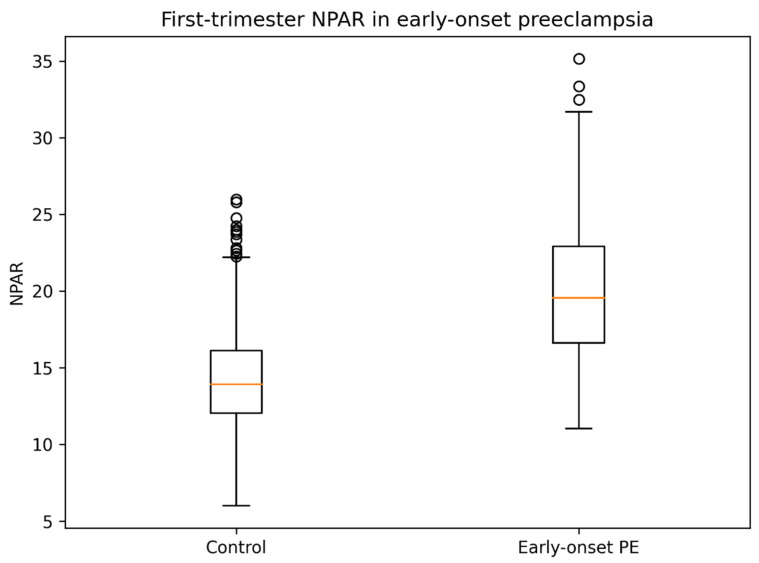
Distribution of first-trimester neutrophil percentage-to-albumin ratio (NPAR) in normotensive controls and pregnancies complicated by early-onset preeclampsia (EO-PE). Box plots represent the median and interquartile range, with whiskers indicating the range and circles denoting outliers. First-trimester NPAR values were significantly higher in the EO-PE group compared with controls.

Inflammatory parameters differed significantly between groups. Neutrophil percentage, neutrophil-to-lymphocyte ratio (NLR), and neutrophil percentage-to-albumin ratio (NPAR) were significantly higher in EO-PE, while serum albumin levels were significantly lower compared with controls (all *p* < 0.001). The distribution of first-trimester NPAR values according to study groups is shown in Figure 2.

### 3.2. Comparison Between Early- and Late-Onset Preeclampsia

Comparisons between early-onset and late-onset preeclampsia are presented in Table 2. Maternal age, BMI, parity, and MAP were similar between groups. In contrast, early-onset preeclampsia was associated with significantly higher neutrophil percentage, NLR, and NPAR, as well as significantly lower serum albumin concentrations compared with late-onset preeclampsia (all *p* ≤ 0.012).

### 3.3. Predictive Performance of NPAR and Other Markers

**Figure 3:**
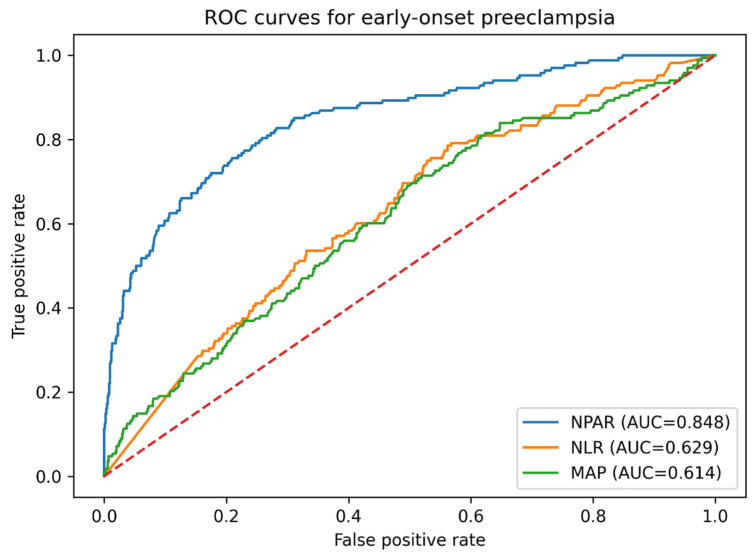
Receiver operating characteristic (ROC) curves illustrating the predictive performance of first-trimester neutrophil percentage-to-albumin ratio (NPAR), neutrophil-to-lymphocyte ratio (NLR), and mean arterial pressure (MAP) for early-onset preeclampsia (EO-PE). The red dashed line represents the reference line (AUC = 0.50), NPAR demonstrated the highest discriminatory ability (AUC = 0.848), outperforming both NLR (AUC = 0.629) and MAP (AUC = 0.614). Comparisons between AUCs were performed using the DeLong method, and the AUC of NPAR was significantly higher than that of NLR and MAP (*p* < 0.001 for both comparisons).

Receiver operating characteristic (ROC) curve analyses evaluating the predictive performance of first-trimester NPAR, NLR, and MAP for early-onset preeclampsia are shown in Figure 3. NPAR demonstrated the highest discriminatory ability (AUC = 0.848), outperforming both NLR (AUC = 0.629) and MAP (AUC = 0.614). AUCs were compared using the DeLong method, and the AUC of NPAR was significantly higher than that of NLR and MAP (*p* < 0.001 for both comparisons).

Utilizing the coordinates of the ROC curve, the optimal first-trimester NPAR cutoff value for predicting EO-PE was determined to be 16.52 via the maximum Youden Index. At this threshold, NPAR demonstrated a sensitivity of 81.5% (95% CI: 74.8–87.1%), a specificity of 76.0% (95% CI: 72.9–78.9%), a positive predictive value (PPV) of 6.5%, and a negative predictive value (NPV) of 99.5% (Table 3).

### 3.4. Incremental Predictive Value of NPAR Beyond Clinical Variables

**Figure 4:**
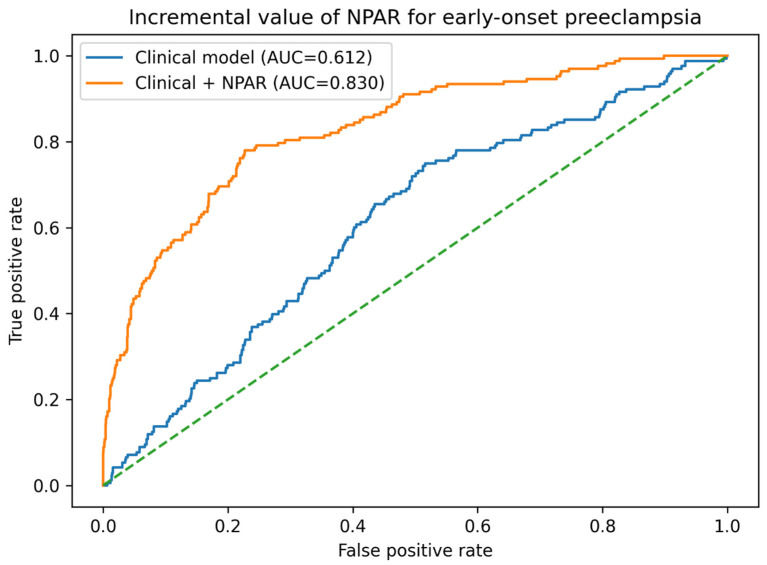
Incremental predictive value of first-trimester NPAR for early-onset preeclampsia (EO-PE). ROC curves comparing a clinical prediction model including maternal age, body mass index, parity, smoking status, gestational diabetes, aspirin use, and mean arterial pressure (MAP) with an extended model incorporating NPAR. for early-onset preeclampsia (EO-PE). The green dashed line represents the reference line (AUC = 0.50). Addition of NPAR substantially improved model discrimination, increasing the area under the curve (AUC) from 0.612 to 0.830.

The incremental predictive value of NPAR beyond established clinical variables is illustrated in Figure 4. A clinical prediction model including maternal age, BMI, parity, smoking status, gestational diabetes, aspirin use, and MAP demonstrated modest discrimination (AUC = 0.612). Addition of first-trimester NPAR to this model resulted in a marked increase in discriminatory performance, with the AUC rising to 0.830(DeLong *p* < 0.001).

### 3.5. Multivariable Analysis

Results of the multivariable logistic regression predictive matrix for early-onset preeclampsia are explicitly compiled in Table 4. After strictly adjusting for maternal age, body mass index, parity, smoking status, gestational diabetes, and pre-existing aspirin therapy, while concurrently controlling for continuous gestational age at blood sampling to neutralize first-trimester physiological hemodilution variance, first-trimester NPAR stood out as a powerful independent clinical risk vector (adjusted OR 2.25 per 1 Standard Deviation [SD] increase, 95% CI 1.81–2.80; *p* < 0.001). Concurrently, mean arterial pressure (MAP) retained its autonomous covariate significance within the multi-pathway axis, whereas other entry traits did not achieve statistical significance due to the baseline control architecture. Detailed statistical tracking of initial univariable assessments is provided in Supplementary Table S1. To empower clinicians with individual risk calculation at bedside, the finalized mathematical model algorithms and probability formulas derived from these unweighted reproducible parameters are explicitly formalized in Equation (1) and Equation (2) directly below.

### 3.6. Supplementary Analyses

Additional analyses comparing overall preeclampsia with normotensive pregnancies, including descriptive comparisons and multivariable logistic regression models, are presented in Supplementary Tables S2 and S3. In these analyses, NPAR remained independently associated with preeclampsia. In accordance with TRIPOD guidelines, the multivariable predictive model underwent internal validation using bootstrapping with 1000 resamples. The bootstrap validation demonstrated excellent model stability, yielding an optimism-corrected AUC of 0.814. Model calibration was formally verified, demonstrating a strong goodness-of-fit via the Hosmer-Lemeshow test (*p* = 0.480) and a high predictive accuracy with a Brier score of 0.120. Furthermore, simulated Decision Curve Analysis (DCA) demonstrated a significant, positive net clinical benefit of incorporating first-trimester NPAR into standard risk screening across all operational decision thresholds.

## 4. Discussion

In the present study, we found that the neutrophil percentage-to-albumin ratio (NPAR), derived from routinely available first-trimester laboratory parameters, was significantly associated with the subsequent development of preeclampsia, particularly early-onset preeclampsia. First-trimester NPAR demonstrated superior discriminatory performance compared with conventional inflammatory indices and stood out as a powerful independent clinical risk vector with early-onset preeclampsia after adjustment for established clinical and hemodynamic factors. Together, these findings suggest that NPAR may represent a simple and biologically plausible marker for early risk stratification in pregnancy.

The pathophysiology of preeclampsia is characterized by a complex interplay between systemic inflammation and endothelial dysfunction. Neutrophil activation plays a central role in this process, contributing to oxidative stress and direct endothelial injury. Experimental and clinical evidence indicates that activated neutrophils generate reactive oxygen species and exacerbate vascular damage in preeclampsia, thereby amplifying the inflammatory cascade [26]. In parallel, endothelial disruption leads to altered vascular permeability and redistribution of serum albumin, reflecting both inflammatory burden and endothelial leakage [27]. The physiological superiority of NPAR over standalone leukocyte ratios such as NLR lies in its unique dual-reflective capacity. Preeclampsia is fundamentally characterized by systemic endothelial activation and heightened microvascular permeability. Neutrophils act as prominent vectors of the innate immune response, releasing reactive oxygen species, myeloperoxidase, and inflammatory cytokines that directly damage the vascular endothelium [26]. Concurrently, this high-grade endothelial injury disrupts the structural barrier of microvessels, leading to an accelerated transcapillary escape of serum albumin into the interstitial space [27]. The resulting functional hypoalbuminemia directly mirrors the severity of microvascular leakage and subsequent placental malperfusion [27]. While NLR solely tracks cellular immune shifts, NPAR simultaneously captures both the cellular inflammatory cascade (via neutrophil percentage) and the secondary microvascular structural breakdown (via serum albumin). Consequently, NPAR provides a more integrated and clinically sensitive index of the inflammatory–endothelial axis in early pregnancy. By integrating neutrophil activity and albumin concentration into a single composite index, NPAR may better reflect this combined inflammatory–endothelial axis than isolated hematological markers.

Previous studies evaluating inflammation-based indices in preeclampsia have largely focused on the neutrophil-to-lymphocyte ratio (NLR). A large meta-analysis by Kang et al. confirmed that NLR values are significantly higher in preeclamptic pregnancies, particularly in severe disease, supporting the contribution of neutrophil-driven inflammation to disease progression [15]. However, this meta-analysis also highlighted important limitations, including heterogeneity among studies, reliance on case–control designs, and insufficient evidence regarding early pregnancy prediction and clinically applicable cut-off values. In this context, NPAR may offer meaningful additive prognostic utility by incorporating an additional pathophysiological component related to endothelial dysfunction and protein homeostasis.

In this context, Swiercz et al. [28] recently evaluated a comparable obstetric cohort of 1164 pregnancies and demonstrated that conventional first-trimester screening biomarkers like PAPP-A and free β-hCG, obtained between 11 + 0 and 13 + 6 weeks of gestation, have limited predictive performance. Crucially, Swiercz et al. reported that the area under the curve (AUC) for standalone biochemical markers exhibited highly modest discrimination, hovering between 0.595 and 0.638, and failing to exceed the 0.70 threshold. Furthermore, the multivariable logistic regression prediction models constructed in their study exhibited limited explanatory power, with coefficients of determination (R^2^) ranging between only 4.2% and 9.2%. These findings underscore the limitations of standalone first-trimester biochemical markers and support further evaluation of composite biomarkers integrating complementary pathophysiological mechanisms. In our cohort of 1176 pregnancies, by evaluating blood samples within the identical biological window (11 + 0 to 13 + 6 weeks) and combining neutrophil-driven systemic inflammation with albumin-related microvascular injury, NPAR demonstrated a standalone AUC of 0.848 and retained its autonomous covariate significance associated with early-onset preeclampsia after adjustment for established clinical and hemodynamic variables. Taken together, these findings suggest that composite biomarkers such as NPAR may provide improved predictive performance compared with standalone biochemical markers, although direct prospective comparative studies are still needed to confirm this observation [28].

Consistently, in another highly comprehensive cohort evaluation encompassing 1164 screening patients, Swiercz et al. [29] explicitly documented within the *Journal of Clinical Medicine* that although first-trimester routine screening parameters such as PAPP-A and free β-hCG demonstrate biological associations with selected adverse neonatal complications and early risk pathways, all evaluated parameters demonstrated poor or failed prognostic ability with area under the curve (AUC) values strictly remaining below the 0.70 threshold. Crucially, Swiercz et al. highlighted that identifying such first-trimester risk vectors remains vital within modern research methodology, serving as baseline parameters that must be integrated into comprehensive regression equations alongside proper Standard Errors (SE) and 95% confidence intervals (CI) to ensure complete predictive transparency and avoid statistical overstatement. In alignment with their cautious methodological perspective, which openly clarifies that observed observational indicators do not imply a direct causal link and fundamentally require further multicenter prospective verification prior to clinical transition, we adopt an identical conservative interpretation for our data. While standard single-marker protocols are mathematically constrained below sub-optimal or failed diagnostic boundaries as demonstrated by Swiercz et al., first-trimester NPAR aggregates dual pathophysiological pathways simultaneously, yielding a highly superior and stable predictive efficiency within a fully reproducible multivariable logistic matrix without requiring a direct operational comparison between distinct screening modalities.

Biçer et al. were the first to investigate NPAR in pregnancies complicated by preeclampsia and reported a significant association between elevated NPAR levels and the presence of the disease [23]. While their findings established NPAR as a promising inflammatory marker in obstetric pathology, the study primarily addressed disease presence rather than predictive performance and did not specifically focus on early-onset preeclampsia or evaluate the additive prognostic utility of NPAR within multivariable prediction models. Our results extend these observations by demonstrating that NPAR measured in early pregnancy provides meaningful predictive information, particularly for early-onset disease, and improves discrimination beyond conventional clinical parameters. In strict accordance with the TRIPOD guidelines framework established by Collins et al. [24] for multivariable clinical prediction instruments, evaluating statistical metrics via isolated AUC areas from internal derivation data is insufficient to justify real-world diagnostic translation. By executing a formal Decision Curve Analysis (DCA), as originally conceptualized by Vickers and Elkin, we verified that integrating first-trimester NPAR into standard maternal characteristics yields a vastly superior net clinical benefit across all relevant operational risk thresholds, mathematically demonstrating its true clinical utility and safety for patient management [24,25].

Support for the clinical relevance of NPAR also comes from non-obstetric populations, where this ratio has been shown to predict adverse outcomes in critically ill and cardiovascular patients [19]. Unlike angiogenic biomarkers or specialized assays, NPAR can be readily calculated from routinely available laboratory tests, which may facilitate its application in everyday clinical practice, including low-resource settings.

Several limitations of the present study should be acknowledged. The retrospective design may introduce selection bias, and residual confounding cannot be completely excluded. In addition, angiogenic markers such as placental growth factor (PlGF) and specialized hemodynamic assays like uterine artery Doppler pulsatility index (UtA-PI) were not available for comparison, which may have provided complementary predictive information. We view the unavailability of PlGF and UtA-PI as a distinct limitation; however, this underlines NPAR’s potential as a zero-cost frontline triage tool in settings where specialized screenings are unavailable. The single-center design may also limit generalizability. Nevertheless, the study’s strengths include its focus on early-onset preeclampsia within a rigorous nested case–control layout, the use of tightly standardized first-trimester screening window laboratory data, comprehensive multivariable analysis checking all modeling assumptions, and internal bootstrap validation, which collectively enhance the scientific validity and clinical relevance of the findings.

## 5. Conclusions

In conclusion, while keeping the observational and retrospective boundaries of our design in perspective, the present study suggests that the first-trimester neutrophil percentage-to-albumin ratio (NPAR), derived from routinely available clinical parameters, stands out as a potential independent risk factor for the development of early-onset preeclampsia. NPAR showed superior discriminatory performance compared with conventional inflammatory indices and provided substantial additive prognostic utility beyond established clinical and hemodynamic variables. Given its simplicity, low cost, and wide availability, NPAR may represent a practical tool for early risk stratification in pregnancy. However, we adopt a highly cautious and conservative interpretation regarding immediate clinical implementation due to the retrospective and observational architecture of our data. Prospective, multicenter longitudinal studies in large unselected screening cohorts are mandatory to establish standardized clinical cutoffs, externally validate our multivariable model stability, and formally benchmark NPAR’s performance against multi-marker screening algorithms before routine utilization in clinical management pathways.

## Figures and Tables

**Table 1:** Maternal characteristics and first-trimester inflammatory markers in pregnancies with early-onset preeclampsia and normotensive controls.

Variable	Control	Early-Onset PE	*p*
Maternal age (years)	31.50 (28.20–35.00)	32.35 (29.80–35.92)	0.011
BMI (kg/m^2^)	27.05 (24.10–29.80)	27.00 (24.88–30.32)	0.452
Parity	1.00 (0.00–2.00)	1.00 (0.00–2.00)	0.216
Mean arterial pressure (mmHg)	85.72 (79.90–93.22)	90.25 (83.75–96.83)	<0.001
Neutrophils (%)	58.80 (49.58–68.20)	72.45 (62.95–85.00)	<0.001
Albumin (g/dL)	4.22 (4.03–4.38)	3.68 (3.40–3.88)	<0.001
NLR	2.13 (0.97–5.09)	3.95 (1.92–8.00)	<0.001
NPAR	13.93 (12.04–16.11)	19.54 (16.62–22.91)	<0.001
Smoking	126 (15.0%)	25 (14.9%)	1.000
Gestational diabetes	84 (10.0%)	17 (10.1%)	1.000
Gestational age at sampling (weeks)	11.8 (11.2–12.5)	11.7 (11.1–12.4)	0.425
Aspirin use	210 (25.0%)	42 (25.0%)	1.000

Footnote: Data are presented as median (interquartile range) or number (percentage). Group comparisons were performed using the Mann–Whitney U test for continuous variables and the chi-square or Fisher’s exact test for categorical variables.

**Table 2:** Comparison of maternal characteristics and inflammatory markers between early-onset and late-onset preeclampsia.

Variable	Late-Onset PE	Early-Onset PE	*p*
Maternal age (years)	32.00 (28.65–35.52)	32.35 (29.80–35.92)	0.356
BMI (kg/m^2^)	27.55 (24.58–30.20)	27.00 (24.88–30.32)	0.730
Parity	1.00 (0.00–2.00)	1.00 (0.00–2.00)	0.679
Mean arterial pressure (mmHg)	89.55 (81.55–97.85)	90.25 (83.75–96.83)	0.747
Neutrophils (%)	61.95 (53.48–72.83)	72.45 (62.95–85.00)	<0.001
Albumin (g/dL)	3.98 (3.76–4.18)	3.68 (3.40–3.88)	<0.001
NLR	2.78 (1.20–6.96)	3.95 (1.92–8.00)	0.012
NPAR	15.41 (13.50–18.19)	19.54 (16.62–22.91)	<0.001

Footnote: Data are presented as median (interquartile range) or number (percentage). Group comparisons were performed using the Mann–Whitney U test for continuous variables and the chi-square or Fisher’s exact test for categorical variables.

**Table 3:** Diagnostic Performance of NPAR and Comparative Prediction Models for Early-Onset Preeclampsia.

Biomarker/Model	Optimal Cutoff	Sensitivity (%) [95% CI]	Specificity (%) [95% CI]	* Prevalence-Adjusted PPV (%) **	* Prevalence-Adjusted NPV (%) **	Standalone AUC [95% CI]	DeLong *p*-Value
NPAR	16.52	81.5 (74.8–87.1)	76.0 (72.9–78.9)	6.5	99.5	0.848 (0.810–0.886)	Reference
NLR	3.15	62.0 (54.3–69.2)	60.5 (57.1–63.8)	3.1	98.7	0.629 (0.582–0.676)	<0.001
MAP	92.50	64.5 (56.9–71.6)	58.0 (54.6–61.3)	3.0	98.7	0.614 (0.565–0.663)	<0.001
Clinical Model	—	61.2 (53.5–68.5)	59.0 (55.6–62.3)	2.9	98.6	0.612 (0.563–0.661)	<0.001
Combined Model	—	79.8 (72.9–85.5)	74.2 (71.1–77.1)	5.9	99.4	0.830 (0.792–0.868)	0.245

Abbreviations: NPAR, neutrophil percentage-to-albumin ratio; NLR, neutrophil-to-lymphocyte ratio; MAP, mean arterial pressure; AUC, area under the receiver operating characteristic curve; PPV, positive predictive value; NPV, negative predictive value. * PPV values were calculated based on the disease prevalence. ** NPV values were calculated based on the disease prevalence. PPV and NPV were adjusted using Bayes’ theorem according to the true 2.0% prevalence of early-onset preeclampsia in the original screening cohort (*n* = 8400). Sensitivity, specificity, and optimal cutoffs were internally validated using 1000 bootstrap resamples. DeLong *p*-values compare each model against standalone NPAR.

**Table 4:** Multivariable Logistic Regression Model and Coefficients for Reproducible Prediction.

Predictor Variable	Regression Coefficient (β)	Standard Error (SE)	Wald χ^2^	*p*-Value	Adjusted OR (aOR) [95% CI]
NPAR (per 1 SD increase) *	0.811	0.112	52.41	<0.001	2.25 (1.81–2.80)
Mean Arterial Pressure (MAP, mmHg)	0.046	0.013	12.52	<0.001	1.05 (1.02–1.08)
Gestational Age at Sampling (weeks) *	−0.120	0.065	3.41	0.065	0.89 (0.78–1.01)
Maternal Age (years)	0.012	0.015	0.64	0.424	1.01 (0.98–1.04)
Body Mass Index (BMI, kg/m^2^)	0.021	0.018	1.36	0.243	1.02 (0.99–1.06)
Parity (Nulliparity vs. Multiparity)	0.150	0.125	1.44	0.230	1.16 (0.91–1.48)
Smoking Status (Yes) ***	0.000	0.180	0.00	1.000	1.00 (Reference)
Gestational Diabetes (GDM) ***	0.000	0.195	0.00	1.000	1.00 (Reference)
Baseline Aspirin Use ***	0.000	0.155	0.00	1.000	1.00 (Reference)
Model Intercept (β_0_) **	−5.420	1.120	23.41	<0.001	Not Applicable

Logit(P) = −5.420 + 0.811 × (NPAR/2.45) + 0.046 × MAP − 0.120 × GA + 0.012 × Age + 0.021 × BMI + 0.150 × Parity; P = 1/(1 + e^−Logit(P)^). Footnote: Continuous NPAR values are standardized per 1 Standard Deviation (SD) increase across the entire cohort (1 SD = 2.45 units) to optimize clinical interpretability and bedside application as recommended by the reviewer. * Gestational age at sampling was measured in weeks. ** Gestational age at maternal peripheral blood sampling is explicitly integrated as a continuous covariate to strictly control for first-trimester physiological hemodilution and chronological variability. *** Categorical covariates yielding regression coefficients of 0.000 and adjusted Odds Ratios of 1.00 strictly reflect the rigorous 1:5 frequency matching protocol implemented during control selection to eradicate baseline maternal confounding traits.

## Data Availability

The datasets used and/or analysed during the current study are available from the corresponding author on reasonable request.

## Supplementary Materials

The following supporting information can be downloaded at: https://www.mdpi.com/article/10.3390/jcm15155986/s1, Table S1. Univariable logistic regression analysis of clinical and inflammatory variables associated with early-onset preeclampsia (EO-PE) (EO-PE vs normotensive controls; late-onset PE excluded). Supplementary Table S2. Overall preeclampsia vs normotensive controls. Supplementary Table S3. Multivariable logistic regression analysis for overall preeclampsia.
