# Supplementary material for: First-Trimester Neutrophil Percentage-to-Albumin Ratio Adds Meaningful Additive Prognostic Utility for Early-Onset Preeclampsia: A Nested Case-Control Study
Source: J Clin Med. 2026 Jul 31;15(15):5986. doi: 10.3390/jcm15155986 (PMC13466379; doi:10.3390/jcm15155986)
Supplement: Supplementary file 1 [file jcm-15-05986-s001.zip › jcm-4420306-supplementary.pdf]

Supplementary Tables

Table S1. Univariable logistic regression analysis of clinical and inflammatory variables associated with early-onset preeclampsia (EO-PE) (EO-PE vs normotensive controls; late-onset PE excluded).

Variable	OR	95% CI	p value
Neutrophil percentage-to-albumin ratio (NPAR)	1.472	1.388–1.561	<0.001
Neutrophil-to-lymphocyte ratio (NLR)	1.348	1.167–1.557	<0.001
Mean arterial pressure (MAP)	1.045	1.027–1.063	<0.001
Maternal age (years)	1.039	1.005–1.075	0.024
Body mass index (kg/m²)	1.018	0.978–1.058	0.385
Parity	1.111	0.947–1.302	0.195
Smoking status (yes vs no)	0.991	0.622–1.577	0.969
Gestational diabetes (yes vs no)	1.013	0.585–1.756	0.963
Aspirin use (yes vs no)	1.000	0.682–1.466	1.000

Footnote:Odds ratios (ORs) are presented per 1-unit increase for continuous variables and as yes vs no for binary variables.

Supplementary Table S2. Overall preeclampsia vs normotensive controls

Variable	Control	Preeclampsia	p
Maternal age (years)	31.50 (28.20–35.00)	32.10 (28.90–35.73)	0.009
BMI (kg/m²)	27.05 (24.10–29.80)	27.20 (24.70–30.23)	0.136
Parity	1.00 (0.00–2.00)	1.00 (0.00–2.00)	0.189
Mean arterial pressure (mmHg)	85.72 (79.90–93.22)	89.73 (82.47–97.68)	<0.001
Neutrophils (%)	58.80 (49.58–68.20)	66.60 (55.80–78.90)	<0.001
Albumin (g/dL)	4.22 (4.03–4.38)	3.86 (3.61–4.09)	<0.001
NLR	2.13 (0.97–5.09)	3.26 (1.31–7.78)	<0.001
NPAR	13.93 (12.04–16.11)	17.09 (14.36–20.27)	<0.001
Smoking	126 (15.0%)	63 (15.0%)	1.000
Gestational diabetes	84 (10.0%)	42 (10.0%)	1.000
Aspirin use	210 (25.0%)	105 (25.0%)	1.000

Footnote: This table presents baseline characteristics and inflammatory indices comparing overall preeclampsia and normotensive pregnancies (provided in the supplementary Excel analysis).

Supplementary Table S3. Multivariable logistic regression analysis for overall preeclampsia

Predictor	aOR (95% CI)	p
MAP	1.04 (1.03–1.05)	<0.001
Maternal_age	1.03 (1.00–1.05)	0.055
BMI	1.02 (0.99–1.06)	0.134
Parity	1.06 (0.94–1.21)	0.338
Smoking	0.99 (0.69–1.44)	0.977
GDM	1.11 (0.71–1.73)	0.651
Aspirin	0.94 (0.69–1.27)	0.687
NPAR	1.28 (1.23–1.33)	<0.001

Footnote: This table shows adjusted odds ratios for overall preeclampsia including NPAR and clinical covariates (provided in the supplementary Excel analysis).
